# DETECTION OF ELDER ABUSE IN JAPAN NOT COVERED BY THE ELDER ABUSE PREVENTION LAW IN COMPARISON WITH WHO DEFINITION

**DOI:** 10.1093/geroni/igac059.1359

**Published:** 2022-12-20

**Authors:** Tadashi Wada, Hitoshi Suda, Kana Satoh

**Affiliations:** Irahara Primary Care Hospital, Matsudo City, Chiba, Japan; SEITOKU University, Matsudo city, Chiba, Japan; Teikyo University of Science, Adachi, Tokyo, Japan

## Abstract

The Elder Abuse Prevention Law mainly defines “elder abuse” inflicted by caregivers. Elder abuse by non-caregivers is therefore rarely covered by the law. We investigated domestic elder abuse reported in Matsudo City, Chiba, Japan, to determine the number of domestic elder abuse cases excluded from legal protection but constituting elder abuse defined by WHO. From April 2017 to March 2020, the municipal elder protection services agency received 525 reports of domestic elder abuse. We studied the effective records of 497 of these cases. 299 cases were confirmed elder abuse cases defined by the law, with 198 excluded. However, 176 out of the 198 excluded cases constitute elder abuse defined by WHO. In these 176 cases, abuse was perpetrated by non-caregivers. Due to the current formation of the law, this abuse went undetected and unprotected. We think amendment of the law is required to protect these excluded cases.

